# Environmental Effects on Temperature Stress Resistance in the Tropical Butterfly *Bicyclus Anynana*

**DOI:** 10.1371/journal.pone.0015284

**Published:** 2010-12-20

**Authors:** Klaus Fischer, Anneke Dierks, Kristin Franke, Thorin L. Geister, Magdalena Liszka, Sarah Winter, Claudia Pflicke

**Affiliations:** 1 Zoological Institute and Museum, University of Greifswald, Greifswald, Germany; 2 Department of Animal Ecology I, University of Bayreuth, Bayreuth, Germany; Smithsonian's National Zoological Park, United States of America

## Abstract

**Background:**

The ability to withstand thermal stress is considered to be of crucial importance for individual fitness and species' survival. Thus, organisms need to employ effective mechanisms to ensure survival under stressful thermal conditions, among which phenotypic plasticity is considered a particularly quick and effective one.

**Methodology/Principal Findings:**

In a series of experiments we here investigate phenotypic adjustment in temperature stress resistance following environmental manipulations in the butterfly *Bicyclus anynana.* Cooler compared to warmer acclimation temperatures generally increased cold but decreased heat stress resistance and vice versa. In contrast, short-time hardening responses revealed more complex patterns, with, e.g., cold stress resistance being highest at intermediate hardening temperatures. Adult food stress had a negative effect on heat but not on cold stress resistance. Additionally, larval feeding treatment showed interactive effects with adult feeding for heat but not for cold stress resistance, indicating that nitrogenous larval resources may set an upper limit to performance under heat stress. In contrast to expectations, cold resistance slightly increased during the first eight days of adult life. Light cycle had marginal effects on temperature stress resistance only, with cold resistance tending to be higher during daytime and thus active periods.

**Conclusions/Significance:**

Our results highlight that temperature-induced plasticity provides an effective tool to quickly and strongly modulate temperature stress resistance, and that such responses are readily reversible. However, resistance traits are not only affected by ambient temperature, but also by, e.g., food availability and age, making their measurement challenging. The latter effects are largely underexplored and deserve more future attention. Owing to their magnitude, plastic responses in thermal tolerance should be incorporated into models trying to forecast effects of global change on extant biodiversity.

## Introduction

The ability to withstand environmental stress is of crucial importance for any species' longer-term survival, and the associated stressors are consequently deemed among the strongest forces of natural selection [1]–[3]. Among the large number of stressors temperature is considered a particularly important one, because variable thermal environments are common and may pose substantial challenges for individual survival and reproduction [4]–[8]. The importance of an individual's temperature stress resistance will further increase in the future due to global warming, causing a raise in mean temperatures but also in temperature extremes, which may comprise the largest anthropogenic challenge ever placed on natural systems [9]–[13].

Given that variation in temperature is all pervasive and that temperature extremes may occur in most ecosystems more or less frequently, organisms need to employ effective mechanisms to ensure survival under such stressful conditions. According mechanisms to adjust phenotypic values to environmental conditions, including behavioural, physiological and molecular ones, are indeed generally found [5], [14]–[15]. Conceptually, they can be categorized into two classes: longer-term genetic adaptation (e.g. through changes in allele frequencies) and phenotypic plasticity [16]–[17]. Phenotypic plasticity, on which we will focus here, comprises environmental effects on phenotypic expression, being either an adaptive strategy to cope with short-term environmental variation, or alternatively a non-adaptive biochemical or physiological interaction of an organism with its environment [16]–[17].

Plastic responses to temperature variation can offer quick and effective means to cope with thermal stress, including, amongst others, rapid hardening and acclimation. Rapid hardening refers to an increased performance under temperature extremes after a brief (typically 1–2 hours) pre-exposure to less extreme temperatures, which has been described in several insect species and some other arthropods, e.g. [18]–[21]. Acclimation, in turn, is defined as a facultative response to changes in a single environmental variable, typically in the adult stage [1], [22]. The difference to hardening is that acclimation typically involves longer periods of time, typically several days. Both mechanisms have been repeatedly found to affect temperature stress resistance in *Drosophila* and some other organisms, e.g. [5], [15], [23]–[29], though still fairly little is known especially for the effects of hardening and acclimation on chill-coma recovery time [30]. Consequently, we mainly focus on cold stress resistance here.

To investigate environmental effects on temperature stress resistance, we here use two well-established proxies: chill-coma recovery time (i.e. the time needed to regain mobility following cold exposure) and heat knock-down time (i.e. the time until being knocked down under heat stress). Both indices are considered reliable proxies of climatic cold and heat adaptation, respectively, e.g. [7], [27], [31]–[32]. We have chosen the tropical butterfly *Bicyclus anynana* as model organism for this study for the following reasons: (1) the specific population used here inhabits a seasonal environment with an adverse (cool) dry season and a beneficial (warm) wet season, thus promoting phenotypic plasticity [33], (2) there is a solid knowledge on plastic responses in some life-history traits already, e.g. [25], [34]–[35], and (3) because of its tropical origin [36]. The latter seems important as recent studies suggested that tropical ectotherms, living currently close to their (upper) critical thermal limits already, may be particularly vulnerable to global warming [13], [37], and may further show very limited evolutionary potential to respond to future climate change [38]–[39].

In a series of experiments we here address the following research questions: 1) Does temperature stress resistance in *B. anynana* respond to acclimation temperature, how long does it take for an acclimation response to occur, and to what extent is this response reversible? 2) Does stress resistance further respond to short time exposure to different temperatures (‘hardening’)? 3) What is the effect of extreme cold stress on the acclimation response? 4) Does cold stress resistance and the ability to acclimate to a novel environment diminish with age? 5) Does the acclimation response in cold stress resistance depend on the specific assay conditions used? 6) Is chill-coma recovery time related to other proxies for fitness such as survival rate? 7) Does larval and adult food stress interfere with the ability to withstand thermal stress? 8) Does temperature stress resistance show variation in relation to daily light cycle?

Assuming that phenotypic changes in temperature stress resistance are adaptive, we predict that warm acclimation and hardening temperatures will increase heat stress resistance, while cool acclimation and hardening temperatures will increase cold stress resistance, e.g. [24]–[26], [28]–[29]. However, how long such responses need to take effect, whether they are readily reversible and to what extent they depend on prior thermal experience has thus far received little attention, while these issues may be of great ecological importance in environments showing strong temperature fluctuations. Another largely open question is whether the ability to acclimate to a novel environment depends on age. If such plastic responses come at any meaningful cost (as is suggested by theory [4], [40]–[41]), older individuals with less resources available should show a decreasing performance with increasing age, e.g. [42]–[43]. Note in this context that butterflies, as other holometablous insects, typically loose mass as they age, indicating resource depletion [44–46). If temperature stress resistance was indeed subject to resource-allocation trade-offs, food stress is also predicted to negatively impact on thermal performance, as is the case for many other traits, but is essentially unknown for temperature resistance traits [47]. Further, resistance traits may show variation in relation to time of day. It might be expected that animals are more cold resistant in the morning where temperature is typically low, thus allowing earlier activity, but more heat resistant in the early afternoon where temperature may reach stressfully high values. Low-altitude populations of *Drosophila buzzatti*, for instance, show diel shifts in high-temperature resistance, which is controlled by a circadian rhythm in order to synchronize highest resistance with peak activity ([48]; see also [49]). Finally, two of the questions addressed above (5 & 6) concentrate more on methodological issues. When using chill-coma recovery time as a proxy for cold stress resistance, one might argue that the conditions used to induce a chill coma may include highly artificial settings. We therefore decided to use a range of different temperatures and exposure times in order to explore its effects on the patterns found. Further, the adaptive significance of a shorter recovery time is not always straightforward, although it correlates with differences in the thermal niche occupied [50] as well as geographical variation in cold stress resistance, e.g. [31], [51]–[52]. Therefore, we test whether cold-acclimated animals showing shorter recovery times also show increased survival rates following cold exposure.

## Materials and Methods

### Study organism

*Bicyclus anynana* is a tropical, fruit-feeding butterfly ranging from Southern Africa to Ethiopia [36]. It exhibits striking phenotypic plasticity with two seasonal morphs, which functions as an adaptation to alternate wet-dry seasonal environments and the associated changes in resting background and predation [53]–[54]. Reproduction in this butterfly is essentially confined to the warmer wet season when oviposition plants are abundantly available, and where 2–3 generations occur. During the colder dry season reproduction ceases and butterflies do not mate before the first rains at the beginning of the next wet season [53], [55]. Laboratory stock populations were established at Bayreuth University, Germany, in 2003, and at Greifswald University, Germany, in 2008, both from several hundred eggs derived from a well-established stock population at Leiden University, The Netherlands. The Leiden population was founded in 1988 from 80 gravid females caught at a single locality in Malawi. In each generation several hundred individuals are reared maintaining high levels of heterozygosity at neutral loci [56]. For this study butterflies from either the Bayreuth or the Greifswald stock population were used.

### Experimental design

For all experiments outlined below, *B. anynana* eggs were collected from several hundred stock females and reared at either 20°C or 27°C (depending on experiment and for purely logistic reasons), high relative humidity (70±10%), and a photoperiod of L12:D12. The temperatures chosen reflect the daily highs in the butterfly's natural environment during the dry and wet season, respectively [53]. Larvae were reared in population cages and fed on young maize (*Zea mays*) plants ad libitum (except for *experiment 8*; see below). Pupae were collected daily and transferred to cylindrical hanging cages, which were checked daily for eclosed butterflies. On their eclosion day, butterflies were randomly allocated to different adult treatment groups as outlined below, except for *experiment 9*, where individuals were allocated to treatments as young larvae already (see below). Thus, except for *experiment 9*, larvae were always reared in a common environment. Unless otherwise stated (cf. *experiment 8*), adult butterflies were fed with moist banana ad libitum.

For measuring chill-coma recovery time, butterflies were placed individually in small translucent plastic cups (125 ml), which were arranged on a tray in a randomized block design. The tray was then exposed to the cold, usually using 19 h at 1°C (for exceptions see below). This method proved to be successful in an earlier study [25]. After cold exposure, trays were transferred to an environmental cabinet with a constant temperature of 20°C. Recovery time was defined as the time elapsed between taking the tray out of the cold until a butterfly was able to stand on its legs. Butterflies were observed for a maximum of 60 min, and this maximum value was used for all animals that had not yet recovered (excluding those few animals from subsequent analyses would not change any of the results presented here qualitatively). To determine heat knock-down time, butterflies were placed in small, sealed glass vials (40 ml), which were submerged in a water bath or transferred to a climate cabinet (Sanyo MIR-553), both set at a constant temperature of 45°C (again in a randomized block design). Note that heating rates may differ between the water bath and the climate cabinet. This, however, does not confound any result shown, as always the same method (either water bath or climate cabinet) was used within one experiment. Butterflies were continuously monitored and heat knock-down time (defined as the time until a butterfly was no longer able to stand upright) for each individual was recorded. Throughout, there was no re-use of any butterflies, i.e. each butterfly was tested only once. In total nine different experiments were carried out. Differences in experimental designs stem partly from follow-up experiments and thus previous results, partly from logistic reasons (i.e. the butterfly numbers available).

#### Effects of acclimation and hardening temperature on cold stress resistance

To investigate effects of acclimation and hardening temperature on cold stress resistance, five different experiments were carried out as detailed below (for an overview of all experiments see Table 1). In *experiment 1*, butterflies were randomly divided among four treatment groups in order to assay effects of acclimation temperature and the reversibility of the acclimation response: exposure for 3 days to 20°C, exposure for 3 days to 27°C, exposure for 3 days to 20°C followed by 3 days at 27°C, and exposure for 3 days to 27°C followed by 3 days at 20°C. Consequently, butterflies were tested on day 3 or 6 of adult life. We predicted that cold-acclimated individuals show shorter recovery times than warm-acclimated ones and that this plastic response is reversible.

**Table 1:** Overview over all experiments carried out.

Experiment	Groups	Factors	Treatments	Dependent variables
**1**	4	Acclimation treatment	3 d at 20°C, 3 d at 27°C, 3 d at 20°C followed by 3 d at 27°C or 3 d at 27°C followed by 3 d at 20°C	CCRT after 19 h at 1°C
**2**	2	Acclimation temperature	3 d at 20°C or 27°C	CCRT after 19 h at 1°C, 50 min at −3.5°C, 90 min at −3.5°C, 45 min at −5°C or 15 min at −8°C; survival after 24 h
**3**	8	Acclimation treatment	Acclimation on three consecutive days to: 20-20-20°C, 27-20-20°C, 20-27-20°C, 27-27-20°C, 20-20-27°C, 27-20-27°C, 20-27-27°C or 27-27-27°C	CCRT after 19 h at 1°C
**4**	16	Acclimation treatment & Age	Four acclimation groups acclimated to 20°C, 27°C, 27°C followed by 20°C or 20°C followed by 27°C; each tested after 2, 4, 6 and 8 d	CCRT after 19 h at 1°C
**5**	5	Hardening temperature	1 h at 6°C, 13°C, 20°C, 27°C or 34°C	CCRT after 19 h at 1°C or after 4 min at −20°C
**6a**	9	Acclimation & Hardening temperature	Factorial design with 3 acclimation (2d) and 3 hardening temperatures (1 h, with 20°C, 27°C, 34°C each)	HKDT at 45°C
**6b**	3	Hardening temperature	1 h at 20°C, 27°C or 34°C	HKDT at 45°C
**6c**	2	Hardening temperature	1 h at 20°C or 39°C	HKDT at 45°C
**7a**	4	Acclimation treatment	6 d at 20°C, 6 d at 27°C, 6 d at 20°C with cold stress on day 3, 6 d at 27°C with cold stress on day 3	HKDT at 45°C
**7b**	2	Acclimation temperature	24 h at 20°C or 27°C	HKDT at 45°C
**8**	4	Larval & Adult food stress	Factorial design with 2 larval and 2 adult feeding treatments (stress versus control each)	CCRT after 19 h at 1°C or HKDT at 45°C
**9**	6	Time of day	Tests at 6 different times of day	CCRT after 19 h at 1°C, CCRT after 20 min to −5°C or HKDT at 45°C

Given are number of experiment, number of treatment groups (excluding sex), factors (also excluding sex), a short description of the treatments involved, and the dependent variables and how they were measured. For details see Experimental design. d: day; CCRT: chill-coma recovery time; h: hour; min: minute; HKDT: heat knock-down time.

In *experiment 2*, butterflies from two acclimation groups (3 days at 20°C or 27°C) were compared for acclamatory responses using different (largely arbitrarily chosen) methods to induce a chill coma, namely 19 h at 1°C, 50 min at −3.5°C, 90 min at −3.5°C, 45 min at −5°C, and 15 min at −8°C. This experiment was designed to investigate whether the acclimation response depends on assay conditions. After having measured chill-coma recovery time, butterflies were transferred to a climate cabinet set at an intermediate temperature of 23.5°C, and survival was recorded 24 h later. We here tested the hypothesis that cold-acclimated butterflies show a higher cold tolerance than warm-acclimated ones regardless of assay conditions.

To further investigate how quickly butterflies with different thermal histories are able to respond to acclimation temperature, eight treatment groups were used in *experiment 3*. Butterflies were acclimated on three consecutive days to different combinations of 20°C and 27°C prior to testing: 20-20-20°C (i.e. acclimated for 3 days to 20°C; treatment 1), 27-20-20°C (acclimated for 1 day to 27°C, followed by 2 days at 20°C; treatment 2), 20-27-20°C (3), 27-27-20°C (4), 20-20-27°C (5), 27-20-27°C (6), 20-27-27°C (7), 27-27-27°C (8). Afterwards, chill-coma recovery time was measured after exposing the butterflies for 19 h to 1°C. We hypothesized that the final day prior to testing has the largest impact on cold resistance, while earlier thermal experience may have some subtle, modulating impact.

*Experiment 4* addressed whether the ability to acclimate to a novel environment diminishes with age. Therefore, butterflies from four treatment groups were tested at days 2, 4, 6, and 8 of adult life. Two control treatment groups were exposed permanently to 20°C and 27°C, respectively. In the other two treatment groups, butterflies were first exposed to the one, but for the last two days prior to testing to the alternative acclimation temperature (treatments 27-20°C and 20-27°C, respectively). Note that in the transfer treatments the ‘day 2’ group did consequently not experience an acclimation temperature change, as butterflies were exposed to the ‘second’ temperature on their eclosion day already. For the other groups, butterflies were exposed for 2 days to 20°C and 2 days to 27°C (day 4 group), for 4 days to 20°C and 2 days to 27°C (day 6 group), and for 6 days to 20°C and 2 days to 27°C (day 8 group, for the 20-27°C treatment) and vice versa (for the 27-20°C treatment). For all individuals used here, pupal mass was measured on day 2 after pupation. After weighing, pupae were kept singly in translucent plastic cups (125 ml) until adult eclosion, after which all butterflies were individually marked for future reference. We here tested the prediction that the ability to acclimate to a novel environment diminishes with increasing with age.

*Experiment 5* investigated the response to different ‘hardening’ temperatures. Butterflies maintained at 27°C were exposed for one hour to 6°C, 13°C, 20°C, 27°C or 34°C. Thereafter, butterflies were either immediately exposed for 19 h to 1°C, or back-transferred to 27°C for one hour for recovery. The latter group was thereafter exposed for 4 min to −20°C (in order to quickly induce a chill coma) prior to measuring chill-coma recovery time. This treatment was included as it appeared questionable whether hardening effects (1 hour exposure) would be measurable after a long-term exposure (19 h) to the cold. Note that, of course, butterflies will not equilibrate to −20°C within 4 minutes and that the temperatures experienced by the butterflies are unknown. However, this matter should be irrelevant for comparing different hardening groups, as conditions were identical across treatment groups and the specific method used to induce a chill coma seems not crucial for such comparisons (see results of experiment 2). We predicted that colder hardening temperatures increase but warmer hardening temperatures decrease cold resistance.

#### Effects of acclimation and hardening temperature on heat stress resistance

Two experiments focussed on temperature effects on heat stress resistance. *Experiment 6* used a full-factorial design with three acclimation and three ‘hardening’ temperatures. Butterflies were acclimated to 20°C, 27°C or 34°C for two days, after which they were divided among the same three temperatures for 1 hour (short time acclimation, here referred to as ‘hardening’). After another two hours at the respective acclimation temperature for recovery, butterflies were tested for heat knock-down time. As no effect of hardening temperature was found using the above design (see below), the experiment was repeated using only one acclimation temperature (27°C, for logistic reasons), from which butterflies were once again exposed for one hour to 20°C, 27°C or 34°C, but tested immediately thereafter (i.e. without a recovery period). Additionally, butterflies (only females, for logistic reasons) acclimated to 27°C were exposed for one hour to 20°C or a more extreme temperature of 39°C, and tested immediately thereafter. We hypothesized that warmer acclimation and hardening temperatures increase heat tolerance and vice versa.

In *experiment 7* the effect of a longer-term cold exposure on the acclimation response was investigated using four treatment groups. Butterflies were acclimated for six days to either 20°C or 27°C. Per acclimation temperature, half of the individuals was exposed for 19 h to 1°C on day 3 of adult life, and afterwards back-transferred to their original acclimation temperature. The other half remained at the respective acclimation temperature throughout (control). We additionally tested whether one day is sufficient to induce a significant acclimation response, by dividing butterflies (only females, again for logistic reasons) reared and maintained at 27°C among 20°C and 27°C for 24 h on day 2 of adult life, prior to investigating heat stress resistance. We here test the prediction that severe cold stress will decrease subsequent heat stress resistance.

#### Effects of larval and adult food limitation on temperature stress resistance

In *experiment 8* a full-factorial design was used to investigate the effects of larval and adult food limitation on temperature stress resistance. Larvae were reared at 27°C and either fed ad libitum (larval control) or starved two-times for 24 h by removing any food from the rearing cages, with one day of food access in-between. To proof that the starvation treatment was successful in imposing stress, pupal mass was measured for all individuals on day 2 after pupation. Upon adult eclosion, butterflies were once again allocated to either a control (fed with banana ad libitum) or a starvation treatment (having access to water only). Temperature stress tolerance was assessed on day 3 of adult life. The experiment consisted of two parts, one addressing effects on chill-coma recovery (A), the other on heat knock-down time (B). Both larval and adult food stress were expected to decrease temperature stress resistance.

#### Effects of light cycle on temperature stress resistance

In *experiment 9* we investigated effects of the time of day on temperature stress resistance. Therefore, we used six climate cabinets (Sanyo MLR-351H) set at 27°C, 70% relative humidity, and a photoperiod of L12:D12. Thus, conditions were the same throughout except for the onset of the light phase, starting at 10 a.m., 2 p.m., 6 p.m., 10 p.m., 2 a.m. or 6 a.m. All individuals were allocated to treatments as young larvae, i.e. ca. 20 days prior to testing temperature stress resistance. This design enabled us to test individuals from all treatments at the same time using a randomized block design. However, the test time equalled different time points with respect to the onset of the light phase from the butterflies' perspective. The experiment consisted of three parts, investigating chill-coma recovery time after exposure for 19 h to 1°C (A), chill-coma recovery time after exposure for 20 min to −5°C (B), or heat knock-down time at 45°C (C). During chilling animals were kept in the dark. Part B was included as it seemed questionable whether effects of photoperiod would be measurable after 19 h in a common environment, after which recovery time was eventually assayed. Butterflies were tested on day 2 (A, C) or 4 (B) of adult life. For part A butterflies were transferred to the cold at 3 p.m., allowing to measure recovery time at 10 a.m. the following day, while for parts B and C exposure to extreme temperatures started at 10 a.m. throughout. We hypothesized that butterflies are more cold-tolerant early in the morning, but more heat-tolerant in the early afternoon.

### Data analyses

Effects of treatment (which may consist of up to two full factors; e.g. acclimation and hardening temperature) and sex on stress resistance traits were analysed using full-factorial AN(C)OVAs, including all factors as fixed effects. Pupal mass was added as covariate in *experiments 4* and *8*. In order to meet ANOVA requirements, data were transformed as appropriate. To standardize between blocks, all stress resistance data were adjusted to block means prior to analysis. Pair-wise comparisons were performed employing Tukey's HSD. Survival data in *experiment 2* were analyzed with nominal logistic regressions on binary data (dead or alive). When comparing two groups only, the t-test (*experiment 6*) or the Mann-Whithney U test (*experiment 7*, as the requirements for parametric testing were not met) was used. All statistical analyses were done using JMP version 4.02 (SAS Institute, 2000) or Statistica 6.1 (StatSoft, 2003). Throughout, all means are given ±1 SE.

## Results

### Effects of acclimation and hardening on cold stress resistance

#### Experiment 1

**Figure 1:**
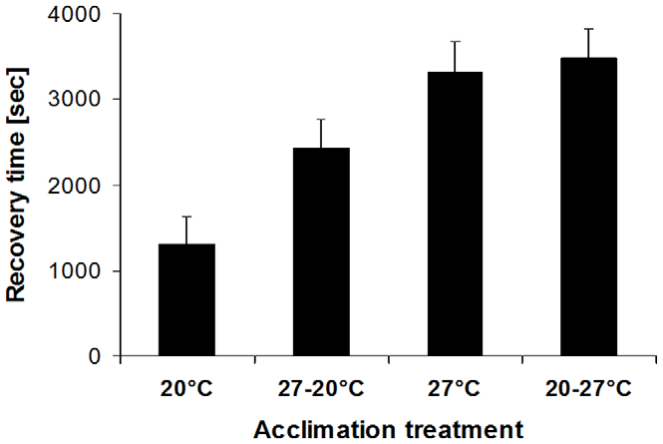
Chill-coma recovery time of *Bicyclus anynana* in relation to acclimation treatment. Butterflies reared in a common environment were acclimated for 3 days to 20°C (20°C), 3 days to 27°C followed by 3 days to 20°C (27-20°C), 3 days to 27°C (27°C), or for 3 days to 20°C followed by 3 days to 27°C (20-27°C). We predicted that cold-acclimated individuals show shorter recovery times than warm-acclimated ones and that this plastic response is reversible. Chill-coma recovery time varied significantly across acclimation groups (F_3,117_ = 11.7, p<0.001; 20°C<27-20°C<27°C = 20-27°C; Tukey HSD after ANOVA), with predictions being largely met. Given are means +1 SE. Sample sizes range between 28 and 34 per group.

Chill-coma recovery time varied significantly across acclimation groups (F_3,117_ = 11.7, p<0.001), but not between sexes (F_1,117_ = 2.9, p = 0.090; treatment by sex interaction: F_3,117_ = 0.2, p = 0.909). Three-day old butterflies exposed to 20°C showed the shortest recovery time, followed by the group exposed first to 27°C but afterwards to 20°C, while both groups exposed to 27°C before testing showed considerably longer recovery times and were statistically indistinguishable (20°C<27-20°C<27°C = 20-27°C; Tukey HSD after ANOVA; Fig. 1).

#### Experiment 2

Using five different methods with a temperature range between +1°C and -8°C and exposure times between 15 min and 19 h yielded a consistent pattern of shorter recovery times for animals acclimated to 20°C compared to 27°C (Table 2A). Consequently, the general pattern of such acclamatory responses is largely independent of the method used to induce a chill coma. Moreover, another proxy for cold stress resistance, mortality rate measured 24 h after cold exposure, invariably revealed a better performance for 20°C- compared to 27°C-acclimated butterflies (n.s. in one case; Table 2B).

**Table 2:** Chill-coma recovery time and mortality rates in *Bicyclus anynana*.

(A) Treatment	20°C [sec]	27°C [sec]	*P*
19 h at 1°C	1547±114	2427±114	**<0.0001**
50 min at −3.5°C	1350±49	1496±49	**0.0402**
90 min at −3.5°C	1312±45	1789±45	**<0.0001**
45 min at −5°C	1198±55	1352±55	**0.0354**
15 min at −8°C	530±55	686±52	**0.0145**

(B) Treatment	20°C [%]	27°C [%]	*P*
19 h at 1°C	15.3	43.5	**0.0128**
50 min at −3.5°C	5.0	25.0	**0.0105**
90 min at −3.5°C	35.0	55.0	0.0696
45 min at −5°C	27.5	50.0	**0.0342**
15 min at −8°C	2.5	20.0	**0.0053**

Chill-coma recovery time (**A**, means ±1 SE) and mortality 24 h after cold exposure (**B**; in %) for 20°C- and 27°C-acclimated *Bicyclus anynana* butterflies across five induction treatments. Significant p-values, as tested by ANOVAs **(A)** and nominal logistic regressions **(B)**, are given in bold. Sexes differed in one out of ten analyses only, with females (47.5%) showing higher mortality rates than males (10.5%) when exposed for 19 h to 1°C (results not shown). Sample sizes are 39 or 40 throughout, except for recovery times in the final treatment, where sample size is only 18 and 20, respectively.

#### Experiment 3

**Figure 2:**
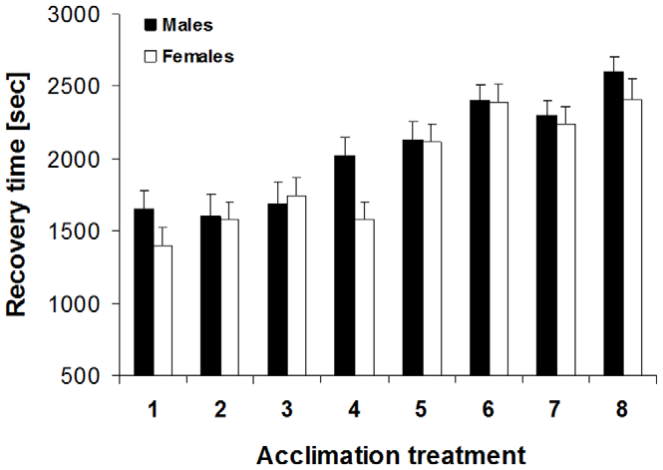
Chill-coma recovery time of *Bicyclus anynana* in relation to sex and acclimation treatment. Butterflies reared in a common environment were acclimated on three consecutive days to different combinations of 20°C and 27°C prior to testing: 20-20-20°C (acclimated for 3 days to 20°C; treatment 1), 27-20-20°C (acclimated for 1 day to 27°C and then for 2 days to 20°C; treatment 2), 20-27-20°C (3), 27-27-20°C (4), 20-20-27°C (5), 27-20-27°C (6), 20-27-27°C (7), 27-27-27°C (8). We hypothesized that the final day prior to testing has the largest impact on cold resistance, while earlier thermal experience may have some subtle, modulating impact. Chill-coma recovery time differed significantly across acclimation groups (F_7,566_ = 19.4, p<0.0001) and sexes (F_1,566_ = 4.9, p = 0.0280), with the thermal environment experienced during the last day prior to testing having the largest impact (1 = 2 = 3 = 4<5<6 = 7 = 8; Tukey HSD after ANOVA). Given are means +1 SE. Sample sizes range between 31 and 46 per group.

In the next experiment we further corroborated that *B. anynana* readily responds to different acclimation temperatures, that this acclimation response is largely reversible, and that the last 24 h prior to testing have the largest impact on stress resistance. Chill-coma recovery time differed significantly across acclimation groups (F_7,566_ = 19.4, p<0.0001) and sexes (F_1,566_ = 4.9, p = 0.0280; interaction: F_7,566_ = 1.0, p = 0.3994). The groups exposed to 20°C during the last 24 h prior to testing showed much reduced recovery times compared to those exposed to 27°C, and females showed shorter recovery times than males (1916±50 sec versus 2045±49 sec; Fig. 2). Note that the most extreme values coincide with permanent exposure to 20°C and 27°C, respectively, while the treatments 27-27-20°C and 20-20-27°C produced fairly intermediate phenotypes.

#### Experiment 4

**Figure 3:**
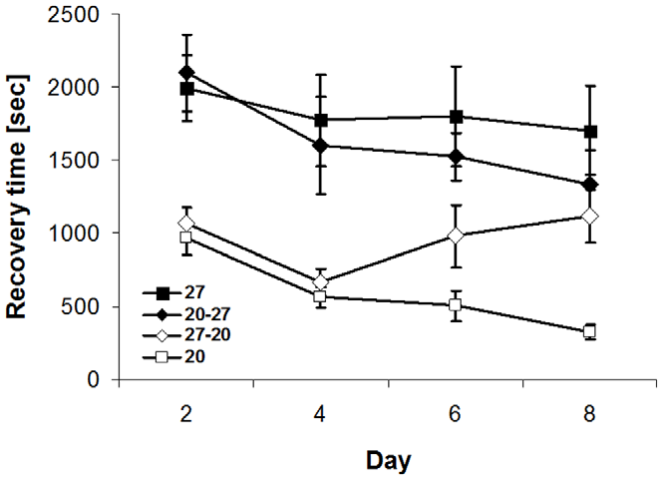
Chill-coma recovery time of *Bicyclus anynana* in relation to acclimation treatment and age. Butterflies reared in a common environment were acclimated for up to 8 days to 20°C, 27°C, 20°C and afterwards 27°C or to 27°C and afterwards 20°C. Butterflies of the ‘transfer groups’ were, depending on age at testing, exposed for 0–6 days to the first, but always for 2 days to the second temperature. Note that accordingly day 2 butterflies did not experience a temperature change. We tested the prediction that the ability to acclimate to a novel environment diminishes with increasing with age, which was not supported by empirical data (interaction between treatment and age not significant; F_1,298_ = 0.7, p = 0.70). Given are means +1 SE. Sample sizes range between 12 and 26 per group.

Following up on the above experiments, the acclimation response was additionally examined in relation to age. Chill-coma recovery time was significantly affected by acclimation treatment, age and the covariate pupal mass (Table 3; Fig. 3). Individuals permanently exposed to 20°C showed the shortest recovery times followed by the group exposed to 20°C during the last two days before testing (27-20°C), while the groups permanently or during the last two days before testing exposed to 27°C needed longer recovery times and were statistically indistinguishable (20°C<27-20°C<20-27°C = 27°C; Tukey HSD after ANOVA, combined for age groups). The age effect indicates that recovery times slightly decreased with increasing age. The non-significant interaction indicates that, within the age classes investigated here, the acclimation response was not negatively affected by age, although recovery times tended to increase at days 6 and 8 for the 27-20°C group (cf. Fig. 3).

**Table 3:** ANCOVA results for the effects of pupal mass (covariate), acclimation treatment and age on chill-coma recovery time. Significant p-values are given in bold.

	DF	MQ	*F*	*P*
**Pupal mass**	1	3882912	4.8	**0.0293**
**Acclimation treatment**	3	26150191	32.3	**<0.0001**
**Age**	3	3312293	4.1	**0.0072**
**Accl. treatment x age**	9	576528	0.7	0.6977
**Error**	298	809584		

#### Experiment 5

**Figure 4:**
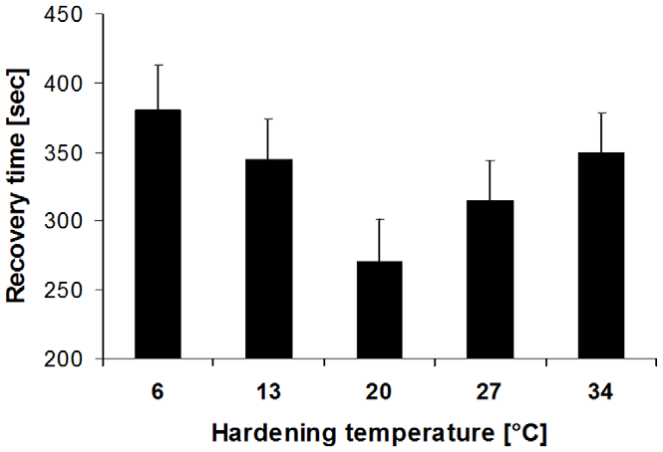
Chill-coma recovery time of *Bicyclus anynana* in relation to hardening temperature. We predicted that colder hardening temperatures increase but warmer hardening temperatures decrease cold resistance. While hardening temperature indeed affected recovery time (F_4,229_ = 3.08, p = 0.0169), both high and low hardening temperatures increased recovery times (6°C = 34°C≥13°C = 27°C (control) ≥20°C; Tukey HSD after ANOVA). Given are means +1 SE. Sample sizes range between 47 and 48 per group.

The response to different hardening temperatures depended on methodology. Exposing butterflies for 19 h to 1°C for measuring chill-coma recovery time yielded no significant effect of hardening temperature (hardening temperature: F_4,227_ = 0.49, p = 0.7473; sex: F_1,227_ = 0.81, p = 0.3701; interaction: F_2,227_ = 1.33, p = 0.2622). Using a four minute exposure to −20°C to induce a chill coma, in contrast, showed that both high and low hardening temperatures significantly increased recovery times (6°C = 34°C≥13°C = 27°C (control) ≥20°C; Tukey HSD after ANOVA; hardening temperature: F_4,229_ = 3.08, p = 0.0169; sex: F_1,229_ = 1.19, p = 0.2763; interaction: F_2,229_ = 0.54, p = 0.7068; Fig. 4).

### Effects of acclimation and hardening on heat stress resistance

#### Experiment 6

**Figure 5:**
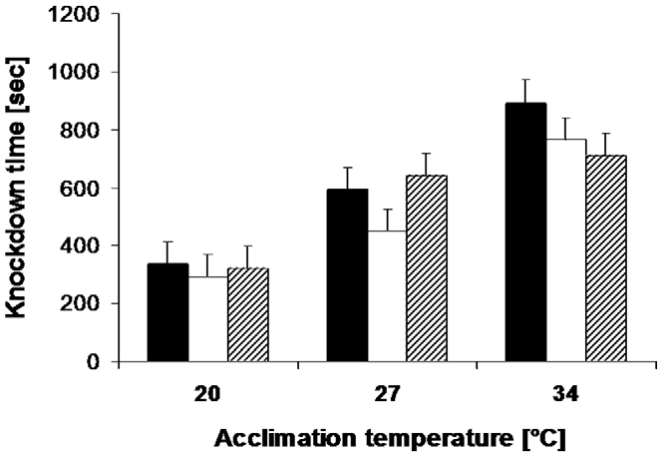
Heat knock-down time of *Bicyclus anynana* in relation to acclimation and hardening temperature. We hypothesized that warmer acclimation and hardening temperatures increase heat tolerance and vice versa. While this prediction was met for acclimation temperature (F_2,321_ = 62.6, p<0.0001; 34°C>27°C>20°C; Tukey HSD after ANOVA), the effect of hardening temperature was not significant (F_2,321_ = 2.2, p = 0.11). Black bars: hardened at 20°C; open bars: hardened at 27°C; hatched bars: hardened at 34°C. Given are means +1 SE. Sample sizes range between 37 and 38 per group.

Heat knock-down time (measured two hours after hardening) was significantly affected by acclimation temperature (34°C>27°C>20°C; Tukey HSD after ANOVA), but not by hardening temperature and sex (Table 4). A significant acclimation by hardening temperature interaction indicates that the differences among acclimation temperatures were rather consistent throughout, except that the individuals acclimated to 27°C and hardened at 34°C showed very similar knock-down times compared to the individuals acclimated and hardened at 34°C (Fig. 5). Testing butterflies acclimated to 27°C immediately after hardening (for 1 hour at 20°C, 27°C or 34°C) once again did not yield a significant effect of hardening temperature on heat knock-down time (hardening temperature: F_2,84_ = 0.94, p = 0.3934; sex: F_1,84_ = 0.30, p = 0.5879; interaction: F_2,84_ = 1.27, p = 0.2871). However, using a more extreme hardening temperature of 39°C revealed a significantly longer knock-down time compared to animals exposed to 20°C (39°C: 561±19; 20°C: 456±19 sec; t_46_ = 4.0, p<0.0001, n = 48).

**Table 4:** ANOVA results for the effects of acclimation temperature, hardening temperature and sex on heat knock-down time.

	DF	MQ	*F*	*p*
**Acclimation T**	2	0.888	62.6	**<0.0001**
**Hardening T**	2	0.032	2.2	0.1080
**Sex**	1	0.021	1.5	0.2202
**Accl. T x Hard. T**	4	0.034	2.4	**0.0478**
**Accl. T x Sex**	2	0.002	0.2	0.8582
**Hard. T x Sex**	2	0.033	2.3	0.0988
**Accl. T x Hard. T x Sex**	4	0.017	1.2	0.3202
**Error**	321	0.014		

Significant p-values are given in bold. T: temperature.

#### Experiment 7

**Figure 6:**
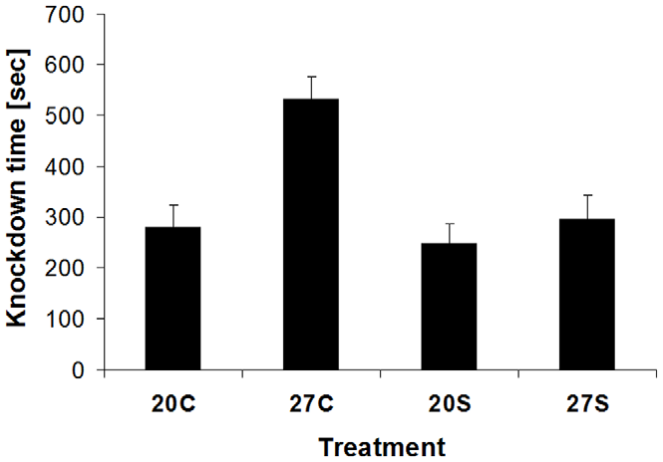
Heat knock-down time of *Bicyclus anynana* in relation to acclimation treatment. Butterflies were acclimated for 6 days to either 20 or 27°C, with half of each acclimation group being subjected for 19 h to 1°C after 3 days. We here test the prediction that severe cold stress will decrease subsequent heat stress resistance, which was confirmed by empirical data (F_3,157_ = 11.2, p<0.001; 27C>27S = 20C = 20S; Tukey HSD after ANOVA). 20C: 20°C control; 20S: 20°C cold-stressed; 27C: 27°C control; 27S: 27°C cold-stressed. Given are means +1 SE. Sample sizes range between 34 and 49 per group.

A clear response to acclimation temperature can be expected within one day already. Butterflies reared and maintained at 27°C, being divided among 20 and 27°C on day two of adult life for 24 h, differed significantly in heat knock-down time with 27°C-acclimated individuals (528±87 sec, n = 20) being much more heat resistant than 20°C-acclimated ones (209±15 sec, n = 20; Mann-Whitney U-test: Z = −3.81, p = 0.0001). However, longer exposure to extreme temperatures may impair the acclimation response. When being subjected for 19 h to 1°C on day three of adult life, butterflies did not regain increased heat stress resistance after a recovery period of another three days at different temperatures: While the 27°C control group showed a significantly increased heat knock-down time, the 27°C group exposed to the cold did not differ significantly from both groups acclimated to 20°C (Tukey HSD after ANOVA; acclimation group: F_3,157_ = 11.2, p<0.001; sex: F_1,157_ = 0.1, p = 0.7146; interaction: F_3,157_ = 0.4, p = 0.7458; Fig. 6).

### Effects of larval and adult food limitation on temperature stress resistance

#### Experiment 8

**Figure 7:**
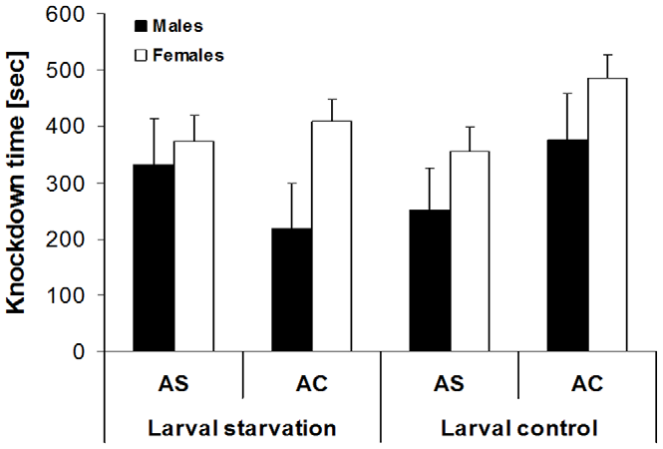
Heat knock-down time of *Bicyclus anynana* in relation to sex, larval and adult feeding treatment. Both larval and adult food stress were expected to decrease temperature stress resistance. Indeed, adult food stress significantly reduced heat tolerance (F_1,227_ = 4.9, p = 0.03), while larval food stress did not (F_1,227_ = 2.2, p = 0.14; though note the significant interaction with adult starvation, cf. Table 5). AS: adult starvation, AC: adult control. Given are means +1 SE. Sample sizes range between 12 and 50 per group.

Food stress during larval development significantly reduced pupal mass (F_1,364_ = 24.2, p<0.001), and females were significantly heavier than males (F_1,364_ = 59.0, p<0.001). As indicated by a significant food stress by sex interaction (F_1,364_ = 6.9, p = 0.009), females were more strongly affected by food stress than males (food stressed males: 136.8±3.7 mg; control males: 143.4±3.0 mg; food stressed females: 151.4±2.4 mg; control females: 173.1±2.2 mg). While chill-coma recovery time was not affected by either factor, heat knock-down time differed significantly across adult feeding treatments and sexes (Table 5). Butterflies deprived of adult food showed reduced knock-down times compared to controls (326±35 sec versus 371±36 sec), and females were more heat resistant than males (406±34 sec versus 326±31 sec; Fig. 7). A significant interaction between larval and adult feeding treatment indicates that lack of adult food greatly reduced heat stress resistance compared to controls in animals fed as larvae ad libitum (303±43 sec versus 431±47 sec), while it tended to increase heat stress resistance in animals having experienced larval food stress (352±47 sec versus 313±45 sec). The latter partly results from the fact that throughout lack of adult food reduced heat knock-down time, except for the males having experienced larval food stress (significant three-way interaction; Fig. 7).

**Table 5:** ANCOVA results for the effects of pupal mass (covariate), larval feeding treatment, adult feeding treatment and sex on chill-coma recovery time (A) and heat knock-down time (B).

(A)	DF	MQ	*F*	*P*
**Pupal mass**	1	1953.6	1.02	0.3142
**Larval starvation**	1	166.6	0.09	0.7684
**Adult starvation**	1	1440.2	0.75	0.3872
**Sex**	1	668.3	0.35	0.5556
**Larv. starv. x adult starv.**	1	6986.4	3.65	0.0583
**Larv. starv. x sex**	1	133.2	0.07	0.7924
**Adult starv. x sex**	1	589.0	0.31	0.5800
**Larv. starv. x adult starv. x sex**	1	467.7	0.25	0.6219
**Error**	123	1912.8		

(B)	DF	MQ	*F*	*P*
**Pupal mass**	1	0.1	0.01	0.9150
**Larval starvation**	1	13.1	2.22	0.1376
**Adult starvation**	1	28.7	4.87	**0.0284**
**Sex**	1	29.2	4.95	**0.0271**
**Larv. starv. x adult starv.**	1	57.1	9.71	**0.0021**
**Larv. starv. x sex**	1	2.9	0.49	0.4838
**Adult starv. x sex**	1	<0.1	<0.01	0.9561
**Larv. starv. x adult starv. x sex**	1	32.8	5.57	**0.0191**
**Error**	227	5.9		

Significant p-values are given in bold.

Note that, regarding the larval by adult feeding interaction, there was a very similar tendency also for chill-coma recovery time (p = 0.058; Table 5A), with a negative effect of adult food stress (prolonged recovery times) in animals having experienced no larval food stress (4477±300 sec versus 3534±254 sec), while cold stress resistance was very similar across adult feeding treatments in animals having experienced larval food stress (3745±405 sec versus 3772±355 sec).

### Effects of light cycle on temperature stress resistance

#### Experiment 9

**Figure 8:**
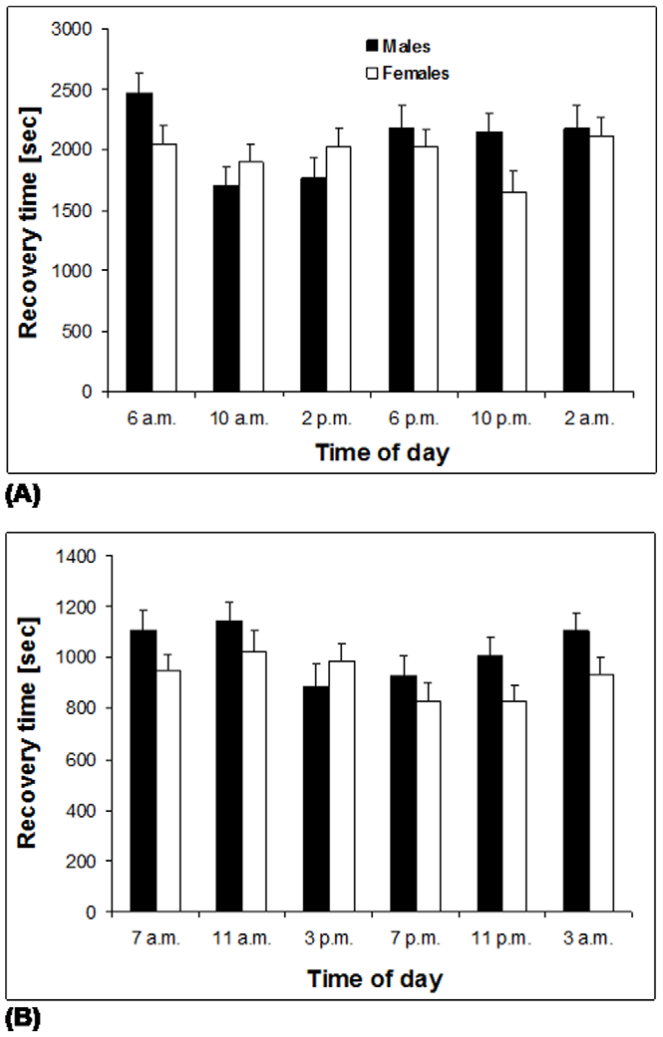
Chill-coma recovery time of *Bicyclus anynana* in relation to time of day and sex. We hypothesized that butterflies are more cold-tolerant early in the morning, but more heat-tolerant in the early afternoon. **(A)** Measured after 19 h at 1°C; **(B)** measured after 20 min at −5°C. In both cases light cycle had a marginally significant effect (F_5,360_ = 2.3, p = 0.045 and F_5,317_ = 2.3, p = 0.046), with cold stress resistance tending to be higher during daytime. The photo phase lasted for 12 h from 6 a.m. to 6 p.m. Given are means +1 SE. Sample sizes range between 22 and 41 per group for **(A)** and between 19 and 35 in **(B)**.

Independent of the method used to induce a chill coma, light cycle had a marginally significant effect on cold stress resistance (Table 6). In the experiment using 19 h at 1°C to induce a chill coma, recovery times tended to be shortest between 10 a.m. and 10 p.m. (Fig. 8A), though note that a post-hoc comparison (Tukey HSD) did not locate any significant differences among groups. The experiment using 20 min at −5°C indicated that chill-coma recovery time was shortest in the afternoon and evening (Fig. 8B), with butterflies tested at 7 p.m. having a significantly shorter recovery time than those tested at 11 a.m. (Tukey HSD after ANOVA; all other pair-wise comparisons n.s.). Further, here females (924±29 sec) needed significantly shorter times to recover than males (1030±32 sec). Heat stress resistance, in contrast, was neither affected by light cycle or sex (Table 6).

**Table 6:** ANOVA results for the effects of light cycle and sex on chill-coma recovery time after 19 h at 1°C (A) or after 20 min at −5°C (B), and on heat knock-down time at 45°C (C).

(A)	DF	MQ	*F*	*p*
**Light cycle**	5	504.4	2.3	**0.0448**
**Sex**	1	341.0	1.6	0.2136
**Light cycle x sex**	5	410.4	1.9	0.0990
**Error**	360	219.6		

(B)	DF	MQ	*F*	*p*
**Light cycle**	5	95.1	2.3	**0.0463**
**Sex**	1	244.6	5.9	**0.0160**
**Light cycle x sex**	5	44.5	1.1	0.3782
**Error**	317	41.7		

(C)	DF	MQ	*F*	*p*
**Light cycle**	5	1518.0	1.1	0.3895
**Sex**	1	443.0	0.3	0.5808
**Light cycle x sex**	5	782.4	0.5	0.7462
**Error**	420	1449.8		

Significant p-values are given in bold.

## Discussion

### Temperature effects on cold stress resistance

Throughout, cool-acclimated butterflies showed a shorter chill-coma recovery time compared to warm-acclimated ones, meaning that the former are more resistant to cold stress than the latter and thus indicating an adaptive response to temperature variation (see below; cf. e.g. [27], [29]–[30]). Such increased performance under stressful temperatures may on the other hand induce non-trivial costs in other traits [4], [30], [57]. The relatively intermediate phenotype of the group first exposed to 27°C and subsequently to 20°C in *experiment 1* (Fig. 1) suggests some carry-over effects of previous thermal experience, which were still measurable after a three-day acclimation period at another temperature. However, a similar tendency was not obvious in the other transfer group, being transferred from 20°C to 27°C, thus questioning the generality of such effects. An alternative explanation would be that the poorer performance of the 27-20°C compared to the 20°C group results from the higher age in the former group (6 versus 3 days). Independent of the lack of consistency in the other transfer group (see above), e*xperiment 4* renders this possibility unlikely, as recovery times decreased rather than increased with age in three out of four groups. Also, a fundamental difference between transfers from warm to cool versus cool to warm is unsupported by additional data (see below, *experiment 3*, but *experiment 4*; cf. [25]).

*Experiment 2* clearly demonstrates that such acclamatory responses are largely independent of the method used to induce a chill coma (see also [30], [52], [58]). We therefore argue that the specific assay conditions used may not be that critical for measuring plastic responses to different thermal environments. The adaptive value of shorter recovery times after cold exposure could be related to an earlier activity in the mornings following relatively cool nights, a longer activity in the evenings and/or generally higher levels of activity at suboptimal temperatures. Consequently, more time would be available for essential behaviours such as foraging, mate location and reproduction. Note in this context that flight performance is strongly related to ambient temperature in butterflies and other ectotherms [59], [60]. Moreover, our data clearly demonstrate that chill-coma recovery time, frequently used as a proxy for cold stress resistance, is closely related to the survival rate after cold exposure [30].

Given that *experiment 1* did not yield conclusive results on the effects of previous thermal experience, this issue was once again addressed in *experiment 3*, using a more sophisticated design. The respective results show that the acclimation response is fairly fast, because the temperature experienced during the last 24 hours prior to testing had a dominating effect on thermal performance (Fig. 2). Although post-hoc comparisons revealed little significant variation except from the clear distinction between the above mentioned groups (i.e. between the animals having experienced 20°C versus 27°C during the last 24 hours prior to testing), the data suggest that the previous thermal environment does have some subtle, modulating effects on cold stress resistance, with effect size increasing with increasing exposure time to an alternative temperature. Results from *experiment 4* corroborate the above data, in showing that the temperature experienced directly before testing has the largest effect on stress tolerance, and that a previous temperature change does modulate the acclimation response to some extent (though significantly only in the 27-20°C group). Further, visual inspection of figure 3 does suggest that the ability to acclimate to another temperature does diminish with longer exposure to a given temperature and thereby age, though this tendency is statistically not supported.

In contrast to expectations, recovery times slightly decreased rather than increased with age (see also [61]). Thus, at least over the age classes tested here, age did not negatively affect recovery times, but rather tended to increase performance. This as well as the lack of interactive effects might be related to the fact that exclusively relatively young butterflies were tested here (note that *B. anynana* may reach substantially longer life spans in the laboratory; [25], [62]). However, we consider this time span as of particular ecological importance, as butterflies (apart from diapausing individuals) typically have fairly short life spans in the field [63]. In any case our data rule out that there is a simple linear decrease in performance with age, which contrasts with results on heat shock survival showing in various insects a strong decline during the early adult period [42], [48]. The few data available for effects of age on chill-coma recovery time to date revealed contradictory evidence and thus no consistent pattern [43], [61].

In addition to the acclimation temperatures discussed above, short-time exposure to different (‘hardening’) temperatures also affected cold stress tolerance (*experiment 5*). However, the response depended on the methodology used to induce a chill coma, as exposure for 19 hours to 1°C did not reveal any significant effect. We assume that the exposure time to 1°C was simply too long in order to yield a measurable effect of a 1 hour exposure to different hardening temperatures. This notion is supported by our additional experiment using a four minute exposure to −20°C following hardening. Here, recovery time was shortest after ‘hardening’ at 20°C, a temperature which is well within the range of temperatures frequently experienced by the butterflies in the field (reflecting the daily highs during the cooler dry season [53], [55]). Interestingly though, cooler as well as warmer temperatures increased recovery times. Thus, higher temperatures negatively affected cold stress resistance as expected, but the same was true for lower temperatures of 6°C and 13°C, with at least the latter certainly occurring occasionally in the field. These data clearly suggest that the latter temperatures are already stressful for this tropical butterfly, thus diminishing rather than improving subsequent performance [22], [25]. Partly similar results regarding negative effects of hardening on chill-coma recovery time were obtained in *Drosophila* [30].

### Temperature effects on heat stress resistance

As with cold stress resistance, acclimation temperature induced a significant effect on heat stress resistance, with heat knock-down time increasing substantially with increasing acclimation temperature: butterflies acclimated to 34°C resisted heat stress roughly twice as long compared to butterflies acclimated to 20°C [27], [64]. These data once again clearly indicate adaptive phenotypic plasticity, in particular since heat coma is typically followed soon by lethal stress levels. Using short-time exposure to the same three temperatures (20°C, 27°C, 34°C; ‘hardening’), in contrast, did not yield a significant effect, independent of whether butterflies were left to recover for two hours or tested immediately after hardening. These findings suggest that the hardening temperatures used were not extreme enough to induce a rapid hardening response, although the same temperatures were effective in inducing an acclimation response. This notion is supported by an additional set of data comparing hardening temperatures of 20°C and 39°C, where a significant effect of one hour exposure showed a significantly better performance of the latter under heat stress (but e.g. [20] for *Drosophila*). Thus, the mechanisms underlying the hardening response may differ fundamentally from those underlying the (longer-term) acclimation response [30], [58], [65]. The hardening response to heat stress appears to be a sort of emergency mechanism, being activated under acute heat stress only [19], [29], [66]. Alternatively, the exposure time of one hour may have been too short to induce a measurable response when using less extreme temperatures, or the time for recovery may have been too short [20]. Further, our results suggest that heat and cold hardening may also differ with regard to underlying mechanisms [29], [65], as the same hardening temperatures (20°C, 27°C, 34°C) elucidated a clear response in cold stress resistance (see above), but not in heat stress resistance.

Similar to cold stress resistance, variation in heat stress resistance can be expected to occur within 24 hours spent at different temperatures. Nevertheless, exposure for many (19) hours to 1°C clearly reduced subsequent heat stress resistance, although butterflies spent three full days at different acclimation temperatures following the cold stress. Consequently, an extreme (cold) stress event yields longer-lasting effects on subsequent heat stress resistance, lasting well beyond periods typically inducing a clear acclimation response. Interestingly, the negative effect was restricted to the group acclimated to 27°C, while there was no obvious response to extreme cold stress across both groups acclimated to 20°C. Thus, cold exposure interfered with acclimation to warmer temperatures, rather than causing a generally diminished performance.

### Food access and temperature stress resistance

Our larval treatment was successful in imposing food stress, as evidenced by a significant reduction in pupal mass by ca. 10%. Similarly, adult food deprivation is known to reduce body mass in *B. anynana* [46]. Despite the clear evidence for a reduction in the amount of resources available to butterflies, cold stress resistance was not affected by larval or adult feeding treatment. Heat stress resistance, in contrast, was negatively affected by adult food stress, but not (directly) affected by larval food stress (cf. [47] for effects of different larval feeding treatments in *Drosophila*). The latter indicates that the resources carried over from the larval stage may not be that crucial for temperature stress resistance (note in this context the non-significant effect of the covariate pupal mass in both analyses). However, this view is challenged by the interactive effect between larval and adult food stress for heat stress resistance. This significant interaction suggests that only the butterflies having experienced neither larval nor adult food stress showed a better performance under heat stress compared to the other three groups. In other words, butterflies having experienced larval food stress could not take any advantage from having access to adult food (Fig. 7; cf. [67] for reproductive traits). These findings suggest that putatively nitrogenous larval resources, lack of which cannot be compensated for in the adult stage, do actually play a crucial role for stress resistance, setting an upper limit to performance under heat stress [47], [67]. Why adult food access actually reduced heat stress resistance in males having experienced larval food stress, thus showing the opposite pattern compared to all other treatment groups, is difficult to explain and might reflect a chance effect of allocation to treatments.

The discrepancy between heat and cold stress resistance, with the former not being affected by any type of food stress, suggests that the mechanisms involved in heat resistance (e.g. the heat shock response [14]) might be more costly than those involved in cold stress resistance [65], [68]–[69]. However, regarding the above larval by adult feeding treatment interaction for heat stress resistance, there was an analogous statistical trend for cold stress resistance (p = 0.058). Here, adult food stress tended to have a negative impact on cold resistance only if individuals had experienced no larval food stress, while all other groups performed comparably well. Thus, these data may suggest that individuals having been challenged during the larval stage were better prepared to handle adult food stress compared to control individuals [35], [70]–[71]. In any case patterns obtained for heat versus cold stress resistance were strikingly different, which again suggests divergent underlying mechanisms [43], [47]. While the basic mechanism underlying heat stress resistance seems to be the heat shock response, cold stress resistance seems to involve several mechanisms including cryoprotectants, antifreeze proteins, glycerol, heat-shock proteins and changes in membrane fluidity and composition [65].

### Light cycle and temperature stress resistance

While heat stress resistance was not affected by light cycle, cold stress resistance did vary significantly across time of day, independently of the method used to induce a chill coma. However, effect size was small and significance marginal only, which may suggest that the patterns found are biologically irrelevant. On the other hand it is striking that two independent experiments using different approaches yielded qualitatively fairly similar patterns. Overall, it appeared that cold stress resistance was slightly higher during daytime, especially in the afternoon/evening, as compared to night time. Such a pattern could on principle be adaptive, as it might enable higher levels of activity during spells of unfavourably cool weather or in the cool dry season (although butterflies seem to rely in the first place on their cryptic coloration here, thus avoiding unnecessary flight [33], [53]).

### Sex differences and effects of pupal mass

In some experiments sex differences could not be investigated due to the exclusive use of females for logistic reasons. However, in a total of 22 statistical analyses sex was included as factor, yielding in 18 cases a non-significant result. In the remaining cases females showed twice a higher cold stress resistance than males, once a higher heat stress resistance, while survival rates after cold exposure were once lower in females than in males. These results indicate that, overall, sexes seem to be equally stress resistant in *B. anynana* [23], [27], [66].

In two experiments the effect of body size on temperature stress resistance was investigated, by including pupal mass as covariate in the statistical models. While two analyses on heat and cold stress resistance, respectively, revealed no significant effect of pupal mass, it did affect cold stress resistance in *experiment 4*. However, this effect was marginal, and a subsequent Pearson correlation between pupal mass and chill-coma recovery time was non-significant (data not shown). Thus, body size is clearly of subordinate importance for temperature stress resistance in *B. anynana*, challenging the common notion of a positive association between stress resistance and body size.

### Conclusions

Having overall found clear evidence for environmentally-induced variation in temperature stress resistance, one crucial question remains: Did our experimental designs resemble natural conditions closely enough to extrapolate from our results to field conditions? Our answer is a tentative ‘yes’. The acclimation temperatures used are definitively within the range of temperatures experienced by *B. anynana* in its natural environment, which is also true for the majority of ‘hardening’ temperatures [33], [53]. This is the main reason why we decided to use relatively mild ‘hardening’ temperatures throughout. Further, the temperature used to assess heat stress resistance (45°C) will be regularly reached during high solar radiation, at least close to the ground. More critically seem to be the assays on cold stress resistance, as the temperatures used to induce a chill coma were necessarily very low, probably largely without the range of temperatures usually experienced by the butterflies in their natural environment. However, our results also document that the patterns obtained are largely independent of the specific assay conditions used. Further, both heat knock-down and chill-coma recovery time seem to be closely related to fitness, as both correlate with survival rates. We therefore argue that both should be considered convenient proxies of temperature adaptation, even if the experimental conditions chosen do not perfectly resemble natural conditions. Overall, the measurement of acclimation responses seems much less susceptible to assay conditions as compared to critical thermal limits [72]. Nevertheless, we need more studies examining the impact and consequences of using more natural versus more artificial settings.

Our results suggest that temperature-induced plasticity in stress resistance is a striking example of adaptive phenotypic plasticity, thus supporting the beneficial acclimation hypothesis, which has been repeatedly challenged over recent years [22], [73]. Plasticity is thus an effective tool to greatly modulate temperature stress resistance within very short periods of time, thus increasing survival probability under temperature stress [19], [31], [57], [74]. This is likely to be true for the vast majority of organisms, while in some specific cases species may exhibit reduced levels of plasticity, thus limiting quick responses to changing thermal environments [27], [38]–[39], [75]. Such species may be exceptionally vulnerable to the impact of global change [75]. Except from extreme events, such plastic responses are further largely and fairly quickly reversible, enabling adequate responses to fluctuating thermal conditions. As plastic responses may thus fine-tune phenotypes to environmental needs including thermal challenges, the potential costs associated with plastic responses or more plastic genotypes remains a recurrent and still largely unresolved issue in evolutionary biology [41]. In any case the species' ability to respond plastically needs to be incorporated into models trying to forecast effects of global change on extant biodiversity [75]–[76]. Further, we suggest that other factors at least potentially affecting plastic responses, such as limited resource availability which may well go hand in hand with climate change due to the ubiquitous impact of man on natural systems, should not be neglected. Especially investigating interactive effects between food availability and temperature challenges should prove to be a fruitful and valuable area for future research. Facing the increasing temperatures at the global scale, investigating genetic but also plastic responses to temperature will be at the forefront of evolutionary and ecological research for some time to come.
